# Golgi-Located NTPDase1 of *Leishmania major* Is Required for Lipophosphoglycan Elongation and Normal Lesion Development whereas Secreted NTPDase2 Is Dispensable for Virulence

**DOI:** 10.1371/journal.pntd.0003402

**Published:** 2014-12-18

**Authors:** Fiona M. Sansom, Julie E. Ralton, M. Fleur Sernee, Alice M. Cohen, David J. Hooker, Elizabeth L. Hartland, Thomas Naderer, Malcolm J. McConville

**Affiliations:** 1 Department of Biochemistry and Molecular Biology, Bio21 Institute of Molecular Science and Biotechnology, University of Melbourne, Parkville, Victoria, Australia; 2 Faculty of Veterinary and Agricultural Sciences, University of Melbourne, Parkville, Victoria, Australia; 3 Department of Microbiology and Immunology, University of Melbourne at the Peter Doherty Institute for Infection and Immunity, Melbourne, Victoria, Australia; Universidade Federal de Juiz de Fora, Brazil

## Abstract

Parasitic protozoa, such as *Leishmania* species, are thought to express a number of surface and secreted nucleoside triphosphate diphosphohydrolases (NTPDases) which hydrolyze a broad range of nucleoside tri- and diphosphates. However, the functional significance of NTPDases in parasite virulence is poorly defined. The *Leishmania major* genome was found to contain two putative NTPDases, termed LmNTPDase1 and 2, with predicted NTPDase catalytic domains and either an N-terminal signal sequence and/or transmembrane domain, respectively. Expression of both proteins as C-terminal GFP fusion proteins revealed that LmNTPDase1 was exclusively targeted to the Golgi apparatus, while LmNTPDase2 was predominantly secreted. An *L. major* LmNTPDase1 null mutant displayed increased sensitivity to serum complement lysis and exhibited a lag in lesion development when infections in susceptible BALB/c mice were initiated with promastigotes, but not with the obligate intracellular amastigote stage. This phenotype is characteristic of *L. major* strains lacking lipophosphoglycan (LPG), the major surface glycoconjugate of promastigote stages. Biochemical studies showed that the *L. major* NTPDase1 null mutant synthesized normal levels of LPG that was structurally identical to wild type LPG, with the exception of having shorter phosphoglycan chains. These data suggest that the Golgi-localized NTPase1 is involved in regulating the normal sugar-nucleotide dependent elongation of LPG and assembly of protective surface glycocalyx. In contrast, deletion of the gene encoding LmNTPDase2 had no measurable impact on parasite virulence in BALB/c mice. These data suggest that the *Leishmania major* NTPDase enzymes have potentially important roles in the insect stage, but only play a transient or non-major role in pathogenesis in the mammalian host.

## Author Summary

Nucleoside triphosphate diphosphohydrolases (NTPDases) are a family of enzymes expressed in many eukaryotes, ranging from single-celled parasites to mammals. In mammals, NTPDases can have an immunomodulatory role, while in pathogenic protists cell-surface and secreted NTPDases are thought to be important virulence factors, although this has never been explicitly tested. In this study we have investigated the function of two NTPDases, termed LmNTPDase1 and LmNTPDase2, in *Leishmania major* parasites. We show that LmNTPDase 1 and LmNTPDase 2 are differentially targeted to the Golgi apparatus and secreted, respectively. A *Leishmania major* mutant lacking the Golgi LmNTPDase1 exhibited a delayed capacity to induce lesions in susceptible mice when promastigote (insect) stages were used to initiate infection, but not when amastigote (mammalian-infective) stages were used. Loss of promastigote infectivity in the LmNTPDase1 null mutant was associated with the synthesis and surface expression of lipophosphoglycan (LPG), with shorter glycan chains and increased sensitivity to complement-mediated lysis. In contrast, a null mutant lacking the secreted LmNTPDase2 did not exhibit any difference in virulence. Our results suggest that *Leishmania major* NTPDases have specific roles in regulating Golgi glycosylation pathways, and nucleoside salvage pathways in the insect stages, but do not appear to be required for virulence of the mammalian-infective stages.

## Introduction

*Leishmania* parasites cause a spectrum of diseases in humans, ranging from localized cutaneous lesions to disseminated mucocutaneous and lethal visceral infections. It is estimated that 1.5 to 2 million new cases of leishmaniasis occur annually and that more than 350 million people are at risk worldwide. Current first-line drug treatments are suboptimal due to high toxicity, cost, requirement for hospitalization and/or the emergence of drug-resistant strains, highlighting the need for the development of more effective therapeutics [1]. *Leishmania* parasites develop as extracellular promastigote stages in the digestive tract of the sandfly vector [2]. Following injection into the mammalian host during a sandfly bloodmeal, promastigotes are phagocytosed by a range of host cells (neutrophils, dendritic cells and macrophages) before differentiating to obligate intracellular amastigote stages that primarily proliferate within the phagolysosome compartment of macrophages. A number of surface molecules, including an abundant lipophosphoglycan (LPG) and several GPI-anchored glycoproteins, have been shown to be important for promastigote survival during these initial stages of infection [3]. In particular, LPG is thought to form a continuous surface glycocalyx that protects the promastigote stages of most *Leishmania* species from complement-mediated lysis and macrophage-induced oxidative stress during phagocytosis [3]–[5]. However, expression of LPG is down-regulated in amastigote stages and neither LPG nor GPI-anchored proteins are required for the long term growth and survival of this stage in macrophages. The potential role of other promastigote and amastigote secreted and surface proteins in the initiation and establishment of infection is less well defined.

A number of protozoan parasites have been shown to express nucleoside triphosphate diphosphohydrolase activities on their cell surface or in the extracellular milieu [6]–[9], and it has been suggested that hydrolysis of nucleotides may play a role in parasite pathogenesis [10]–[12]. Nucleoside triphosphate diphosphohydrolases (NTPDases, CD39_GDA1 protein superfamily) are a family of enzymes defined by the presence of five apyrase conserved regions (ACRs) and the ability to hydrolyze a wide range of nucleoside tri- and di-phosphates [13]. In mammals, surface-expressed NTPDases function in inflammation and immunity, vascular hemostasis and purine salvage [14], while in the intracellular bacterial pathogen, *Legionella pneumophila*, a secreted NTPDase is required for full virulence in a mouse model of disease [15], [16]. In *Leishmania* species, enzyme activity consistent with the presence of one or more surface-located NTPDases has been observed in both *L. amazonensis* and *L. tropica*, two species responsible for cutaneous leishmaniasis [17]–[19]. A number of lines of indirect evidence suggest that this surface NTPDase activity is important for virulence in the mammalian host. Specifically, surface NTPDase activity is elevated in virulent *Leishmania* strains and in the intracellular amastigote form of the parasite [17]–[19]; inhibition of surface NTPDase activity with chromium (III) adenosine 5′-triphosphate complex, reduced promastigote attachment and entry into mouse macrophages [20]; treatment of parasites with an antibody to the human NTPDase CD39 also reduced the interaction of *Leishmania* with mouse macrophages [19]; finally, polyclonal antibodies raised against synthetic peptides derived from the amino acid sequences of a putative *L. braziliensis* NTPDase caused significant cytotoxicity in cultured *L. braziliensis* promastigotes [21]. While these studies suggest roles for NTPDases in parasite nutrition, surface/secreted NTPDases could also contribute to pathogenesis by inducing host cell purinergic receptors. Purinergic receptors are upregulated in macrophages infected with *L. amazonensis* and these receptors display increased sensitivity to activation by nucleoside triphosphates (NTPs). As changes in the levels of extracellular NTPs and NDPs have been shown to alter purinergic receptor activity and the immune response [22], [23], it has been speculated that hydrolysis of host nucleotides by parasite ecto-NTPDases may restrict the immune response and facilitate parasite proliferation.

While these studies suggest NTPDases may function in *Leishmania* virulence and/or be essential for normal growth and development, they have relied heavily on techniques such as anti-NTPDase antibodies and/or chemical inhibition of enzyme activity to investigate the role of NTPDases in host-parasite interaction. Definitive genetic evidence of a relationship between a parasite NTPDase and parasite virulence is lacking. In this study, we show that *L. major* encodes two NTPDases, termed LmNTPDase1 and LmNTPDase2 (abbreviated to NTPD1 and NTPD2), and we generate null mutants in order to investigate their function during infection of mammalian cells. Our findings suggest that NTPD1 is primarily located to the Golgi apparatus, and plays an important role in regulating both the maturation of surface LPG and the capacity of *L. major* promastigotes to initially establish lesions. In contrast, NTPD2 was secreted, and was not required for lesion development, suggesting that its primary role is in the sandfly vector.

## Methods

### Ethics statement

Use of mice in this study was approved by the Institutional Animal Care and Use Committee of the University of Melbourne (ethics number 1212647.1). All animal experiments were performed in accordance with the Australian National Health Medical Research council guidelines (Australian code of practice for the care and use of animals for scientific purposes, 8^th^ Edition, 2013, ISBN: 1864965975).

### Bioinformatic analysis of putative NTPDases

Putative NTPDases were identified by BLAST [24] searching of the available *Leishmania* genomes, with subsequent manual identification of the conserved ACRs [25], [26]. Protein sequence alignments were performed using ClustalW [27], [28]. SMART [29], [30] was used to identify motifs within the protein sequences.

### Parasite strains and culture conditions

*L. major* substrain MHOM/SU/73/5-ASKH was used to create all mutant and transfected lines. Parasites were routinely cultured as axenic promastigotes in Medium-199 (M199, Gibco, Invitrogen, Australia) supplemented with 10% heat-inactivated foetal bovine serum (FBS, Invitrogen) at 27°C or, prior to mouse infection and LPG purification, in SDM-79 medium supplemented with 10% FBS. G418 (Invitrogen, 100 µg mL^−1^) or nourseothricin (Werner BioAgents, Germany, 100 µg mL^−1^) was used as appropriate to maintain selection pressure on parasites transfected with pXGFP+-derived plasmids or pIR1SAT-derived and pXGSAT-derived plasmids, while puromycin (Invitrogen, 20 µg mL^−1^), hygromycin (Boehringer Mannheim, 100 µg mL^−1^) and bleocin (Calbiochem, 10 µg mL^−1^) were used to select transformants during mutagenesis. Lesion amastigotes were isolated by disrupting murine lesions (diameter 5–10 mm) by passage through a 70 µm plastic sieve, followed by passage through a 27 G needle to lyse macrophages and release parasites [31]. Cell debris was removed by slow speed centrifugation (50×*g*, 10 min, 4°C) and the supernatant centrifuged (2000×*g*, 10 min, 4°C) to collect amastigotes. Amastigotes were washed once in PBS and counted using a haemocytometer prior to use in mouse infections.

### Genetic manipulation of *L. major*

Primer sequences used in genetic manipulation are detailed in supporting information (S1 Table). *L. major* NTPDase null mutants were created via sequential homologous gene replacement in a manner similar to that previously described [32], [33]. All *L. major* PCR products described below were obtained by amplification from genomic DNA. To delete *ntpd1*, an 854 bp 5′ untranslated region (UTR) containing a 5′ *Asp*718 site and a 3′ *Xho*I site was amplified, and a 805 bp 3′ UTR region containing a 5′ *Bam*HI and a 3′ *Sac*I site was amplified. These products were then sequentially cloned into the pBluescript II SK vector (Stratagene, CA, USA). Puromycin or hygromycin resistance cassettes were then excised from pXG-PAC and pXG-HYG [34] respectively and cloned into the *Xho*I/*Bam*HI sites. To functionally delete *ntpd2* a 688 bp fragment of the 5′ gene end was amplified with a 5′*Hin*dIII site and a 3′ *Bam*HI/*Eco*RI/linker region, and an 1156 bp 3′ UTR region containing a 5′ *Bam*HI/*Eco*RI/linker region and 3′ *Not*I site was amplified. An overlap PCR was then performed using these PCR products as template and the resultant product cloned into the *Hin*dIII/*Not*I sites of the pBluescript II SK vector (Stratagene, CA, USA). Puromycin and bleocin resistance cassettes were excised from pXG-PAC and pXG-PHLEO [34] respectively using *Bam*HI and *Eco*RI, and cloned into the engineered *Bam*HI/*Eco*RI sites. Deletion mutant constructs were verified by restriction digest profiles and DNA sequencing. Targeting constructs were then excised by *Kpn*I/*Sap*I (*ntpd1*) or *Hin*dIII/*Not*I (*ntpd2*) digest, gel purified and 5 µg of each sequentially electroporated into *L. major* as described previously [35]. Clonal transfectants resistant to both selection drugs were chosen and deletion of the target gene and integration of resistance cassettes confirmed via triplicate PCR. To generate the pIR1SAT-*ntpd1* construct used in chromosomal complementation, full-length *ntpd1* was excised from pXG-LmNTPDase1-GFP using *Bam*HI and cloned into the *Bgl*II site of the pIR1SAT vector [36], [37]. *Swa*I digest was used to excise 5 µg of targeting DNA for electroporation into *L. major* Δ*ntpd1*. Clonal transformants were selected on basis of resistance to nourseothricin and incorporation into the *ssu* locus confirmed by PCR. To create the LmNTPDase-GFP fusion proteins, full length *ntpd* genes were individually cloned into pXG-GFP^+^ [38]. To express the LPG1-mCherry fusion protein, mCherry from pEGFP-mCherry-N1 [39] was amplified with a 5′*Sma*I/*Bgl*II site and 3′*Bam*HI site and cloned into the *Sma*I/*Bam*HI sites of pXGSAT, generating pXGSAT-mCherry. *lpg1* [40] was amplified and then cloned into *Sma*I/*Bgl*II of pXGSAT-mCherry, creating pXG-LPG1-mCherry. The resulting constructs were confirmed via DNA sequencing and electroporated into wild type *L. major* as previously described [35].

### Subcellular localization of LmNTPDase-GFP fusion proteins using immunoblotting and microscopy

Promastigotes were incubated in serum-free media for 24 hours before harvesting by high speed centrifugation (16000×*g*, 5 min). Supernatants were filtered through a 0.45 µM filter to remove intact parasites before supernatant proteins were precipitated with 10% trichloroacetic acid. The pellet and supernatant fractions were analyzed by standard SDS-PAGE and immunoblotting techniques, with LmNTPDase-GFP fusion proteins detected using anti-GFP antibody (clones 7.1 and 13.1, Roche, Germany) at 1∶1000 dilution. For microscopy studies live cells were immobilized on poly-L-lysine coated coverslips. Cells were visualized and images acquired using a Deltavision Elite fluorescent microscope and SoftWorx software.

### Purification and biochemical analysis of LPG

Stationary phase promastigotes grown in SDM-79 supplemented with 10% FBS were harvested by centrifugation and LPG extracted from de-lipidated cells and purified using octyl-Sepharose chromatography, as described previously [41], [42]. The molecular weight of LPG was assessed via SDS-PAGE and silver staining using standard techniques. LPG was depolymerised with 40 mM trifluoroacetic acid (8 min, 100°C) and dephosphorylated with calf intestinal alkaline phosphatase. The repeat units were desalted by passage over a small column of AG 50-X12 (H+) over AG 4-X4 (OH-) (200 µL of each resin, Biorad) and chromatographed by high performance anion-exchange chromatography (HPAEC). The HPAEC system was equipped with a Dionex GP-50 gradient pump, a Carbo Pac PA-1 column (4×250 mm), with a PA-1 guard column and an ED50 integrated pulsed amperometric detector. The system was controlled and data analyzed by Chromeleon version 6.50 software (DIONEX). The eluents used in the system were 75 mM NaOH (E1) and 75 mM NaOH in 250 mM NaOAc (E2). Elution was performed by the following gradient: T_0_ = 0% (v/v) E2; T_5_ = 0% (v/v) E2; T_40_ = 100% (v/v) E2, T_60_ = 100% (v/v) E2, at a flow rate of 0.6 mL/minute. The phosphatidylinositol moiety of purified LPG was released by nitrous acid deamination (0.25 M sodium nitrite in 0.05 M sodium acetate buffer, pH 4.0; incubated at 40°C for 2.5 h), recovered by partitioning into water-saturated 1-butanol and analyzed using liquid chromatography mass spectrometry (LC/MS).

### Peanut agglutinin assay

Washed stationary phase parasites (10^7^ mL^−1^) were incubated with varying concentrations of peanut agglutinin (PNA) in PBS with 1% bovine serum albumin for 30 minutes at room temperature, and non-agglutinated parasites were counted using a haemocytometer (adapted from [43]).

### Serum sensitivity assay

Serum sensitivity assays were performed in a similar manner to those previously described [5]. Stationary phase promastigotes were washed and resuspended in PBS (10^7^ cells in 500 µL PBS with 1 µg mL^−1^ propidium iodide) and incubated with varying concentrations of human sera for 30 minutes. Fluorescence (indicating cell lysis) was then measured by flow cytometry.

### Mouse model of cutaneous leishmaniasis

Virulence in mice was assessed using the tail base model of cutaneous leishmaniasis, as described previously [31]. Female BALB/c mice (6–8 week old, age-matched) were injected subcutaneously at the tail base. Lesion size was assessed weekly and scored 0–4, as described previously [44]. All parasite cell lines were passaged previously in mice to ensure no loss of virulence unrelated to the known genetic mutations. Parasites were re-isolated from mice as described in the “Parasite strains and culture conditions” section.

### Statistical analysis

Unpaired, two-tailed t-tests were performed using Prism GraphPad software (version 6) and a P value less than 0.05 was considered significant. The exception was when more than two parasite strains were compared, in which case a two-way ANOVA, also using Prism GraphPad software, was performed to simultaneously compare the three different groups. A P value less than 0.05 was considered significant when comparing the differences between the three groups.

## Results

### *L. major* encodes two putative NTPDases that are conserved amongst *Leishmania* species

**Figure 1:**
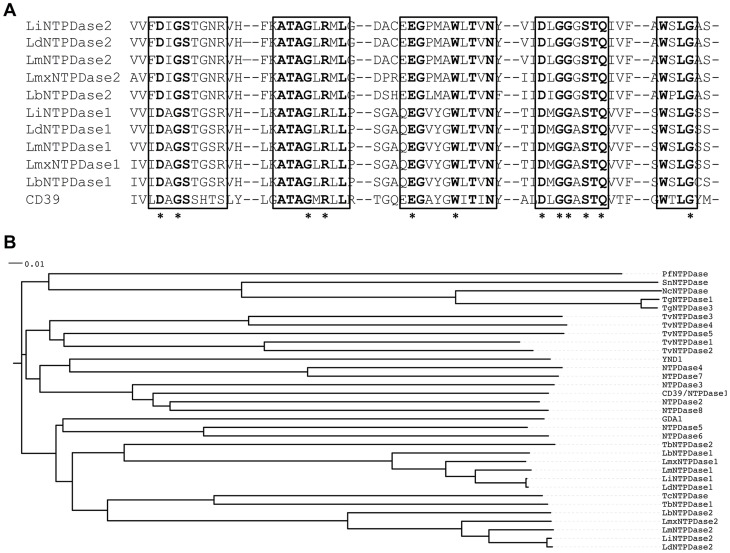
A. Alignment of regions of the putative *Leishmania* NTPDases with human CD39 (NTPDase1). The conserved ACRs are aligned and boxed, with absolutely conserved residues shown in bold. Residues known to be necessary for enzyme function in mammalian NTPDases are starred, revealing all are present in the putative *Leishmania* NTPDases. Alignment was performed using ClustalW [27], [28]. B. Phylogenetic tree of protozoan, yeast and mammalian NTPDases. The tree was constructed from a ClustalW alignment of NTPDase amino acid sequences and viewed and edited using the Interactive Tree of Life web tool [63], [64]. Sequence accession numbers used for Fig. 1A and 1B are given in supplementary S2 Table.

The *L. major* genome contains two putative NTPDase genes (LmjF15.0030 and LmjF10.0170), which are predicted to encode proteins with five ACR domains, the defining feature of all prokaryotic and eukaryotic NTPDase [45]. These genes are conserved amongst all sequenced *Leishmania* species, with homologues present in *L. infantum*, *L. braziliensis*, *L. donovani* and *L. mexicana* [46]. Importantly, a number of residues necessary for enzymatic activity of either CD39 or NTPDase3, the two best characterized mammalian NTPDases [47] are absolutely conserved within the *Leishmania* proteins (Fig. 1A). Using the nomenclature that we previously proposed for the parasite NTPDases [25], we refer to LmjF15.0030 as LmNTPDase1, and Lmj10.0170 as LmNTPDase2 (abbreviated to NTPD1 and NTPD2 in this study for succinctness). Homologues for NTPD1 and NTPD2 are present in *T. brucei*, but only NTPD2 exists in *T. cruzi* (Fig. 1B). Phylogenetic comparison with NTPDases found in other protozoa, mammals and yeast indicates that the trypanosomatid NTPDases are most closely related to mammalian NTPDase5 and NTPDase6, which are usually located intracellularly but can undergo secretion, and to the Golgi-located yeast NTPDase GDA1. Interestingly, the trypanosomatid NTPDases seem evolutionarily distinct from the NTPDases found in a range of apicomplexan parasites and *Trichomonas* protozoa (Fig. 1B), perhaps indicating divergent functions.

### NTPD1 localizes to the Golgi apparatus whereas NTPD2 is secreted from the parasite into the culture supernatant

**Figure 2:**
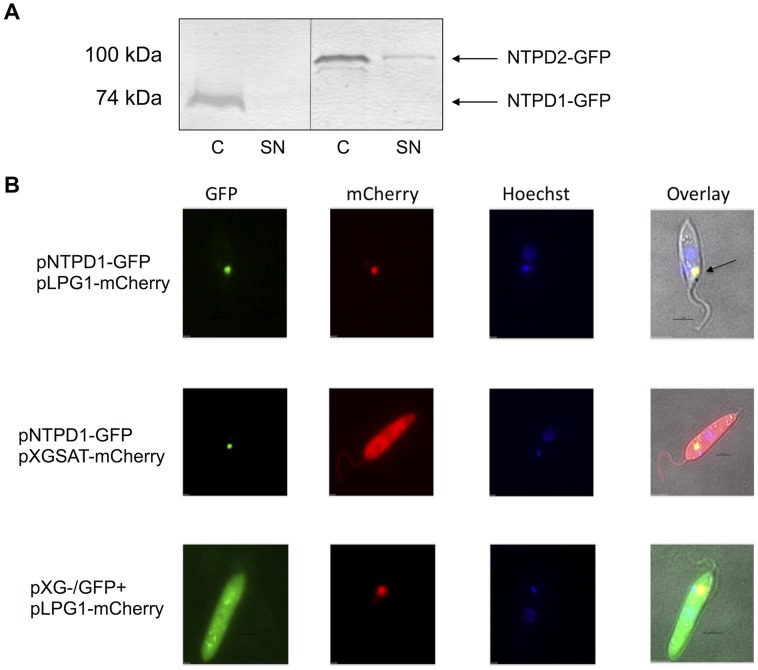
Subcellular localization of LmNTPDase-GFP fusion proteins. A. Western blot using anti-GFP antibody demonstrating production of GFP-fusion proteins of the correct sizes by *L. major* parasites transfected with either pXG-NTPD1-GFP or pXG-NTPD2-GFP, and secretion of NTPD2-GFP into the culture supernatant. Lane 1: *L. major* + pXG-NTPD1-GFP (whole cell lysate, C), Lane 2: *L. major* + pXG-NTPD1-GFP culture supernatant (SN), Lane 3: *L. major* + pXG-NTPD2-GFP C, Lane 4, *L. major* + pXG-NTPD2-GFP SN. Samples were developed simultaneously on one membrane, with the vertical line representing removal of unrelated intervening lanes. B. Localization of NTPD1-GFP to the Golgi apparatus. Top panel: *L. major* co-transfected with pXG-NTPD1-GFP and pXG-LPG1-mCherry; middle panel: *L. major* co-transfected with pXG-NTPD1-GFP and pXG-SAT-mCherry; bottom panel: *L. major* co-transfected with pXG-/GFP+ and pXG-LPG1-mCherry. Arrow indicates co-localisation of NTPD1-GFP and LPG1-mCherry in the Golgi apparatus. Hoechst staining highlights the parasite nucleus (diffuse staining) and kinetoplast (dense staining), with the Golgi apparatus (top and bottom panel, mCherry) in the region adjacent to the kinetoplast (as expected).

*ntpd1* encodes for a protein (432 amino acids) with a putative N-terminal transmembrane domain (residues 17–36), while *ntpd2* encodes for a longer protein (685 amino acids) with an N-terminal signal sequence (residues 1–20). To establish whether the two *L. major* NTPDases are secreted or targeted to the cell surface/intracellular compartment, wild type parasites were transfected with plasmids encoding NTPD1 and NTPD2 as fusion proteins containing C-terminal GFP. Western blot analysis of parasite cell pellets and culture supernatant showed that full-length proteins were expressed in each parasite line (Fig. 2A). Interestingly, while the NTPD1-GFP fusion protein was exclusively associated with the cell pellet, NTPD2-GFP fusion protein was secreted (Fig. 2A). The absence of detectable NTPD1 in the supernatant indicated that the presence of NTPD2 in the culture supernatant was not due to parasite lysis during culture, but represented active secretion (Fig. 2A). Furthermore, live cell fluorescence microscopy of promastigotes expressing NTPD2-GFP did not detect significant cell surface or intracellular fluorescence, consistent with NTPD2 being primarily a secreted protein. Interestingly, Western blot analysis detected a small pool of NTPD2-GFP within the cell pellet fraction (Fig. 2A), which is likely to represent newly synthesized NTPDase in transit to the cell surface, but below the level of detection of fluorescence microscopy. Because of the low abundance of this intracellular pool we can also not discount the possibility that NTPDase2 is directed to other intracellular organelles, such as the lysosome. In contrast, *L. major* promastigotes expressing NTPD1-GFP displayed a single, highly fluorescent punctate stain, at the anterior end of the parasite, proximal to the kinetoplast/flagellar pocket (Fig. 2B). This location is highly characteristic of the Golgi apparatus. *L. major* parasites expressing NTPD1-GFP were therefore co-transfected with a second plasmid encoding the known Golgi protein LPG1 [40] fused to mCherry. Parasites expressing both NTPD1-GFP and the Golgi marker displayed overlapping fluorescence indicative of co-localization (Fig. 2B). This co-localization was not seen in parasites transfected with either mCherry or GFP (both of which display cytoplasmic localization), indicating that NTPD1 is primarily located in the Golgi apparatus. Although yeast NTPDases have been localized to the Golgi apparatus [48], [49], this is the first time a parasite NTPDase has been identified in the Golgi apparatus, rather than being secreted from the parasite or located on the cell surface.

### NTPD1, but not NTPD2, is required for normal lesion development in mice

Previous transcript profiling studies have suggested that *ntpd1* and *ntpd2* are constitutively transcribed in both major developmental stages [50], [51], providing little information on potential stage-specific differences in function. To investigate the function of these enzymes we generated null mutants for each NTPDase gene, by sequential replacement of the two chromosomal alleles with drug resistance cassettes. *ntpd1* was replaced with hygromycin and puromycin resistance cassettes, with gene deletion and correct integration of the resistance cassettes confirmed by triplicate PCR (S1 Fig.), demonstrating that *ntpd1* is not essential under rich culture conditions. In a similar manner *ntpd2* was replaced with puromycin and bleomycin cassettes, with PCR confirmation performed in triplicate (S1 Fig.), indicating that *ntpd2* is also not essential *in vitro*. Both strains grew normally in routine culture medium.

**Figure 3:**
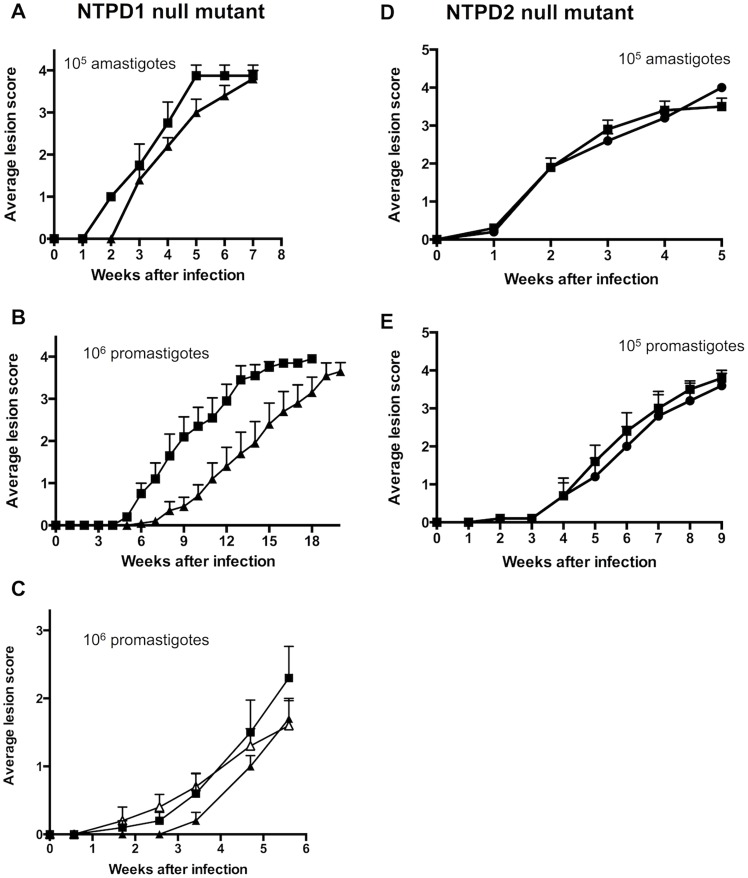
Subcutaneous infection of BALB/c mice with either amastigote (A, D) or promastigote (B, C, E) *L. major*. A. Mice were infected with either 10^5^ wild type *L. major* (squares) or 10^5^ *L. major* NTPD1 null mutant (triangles) amastigotes and lesion scores monitored weekly. Error bars represent S.E.M. (*n* = 5). No significant difference in lesion size was observed at any time point (*P*>0.05, unpaired t-test). B. Mice were infected with either 10^6^ wild type *L. major* (squares) or 10^6^ *L. major* NTPD1 null mutant (triangles) parasites and lesion scores monitored weekly. Error bars represent S.E.M. (*n* = 10). Significant differences in lesion size were observed at all time points from week 6 inclusive (*P*<0.05, unpaired t-test). C. Mice were infected with either 10^6^ wild type *L. major* + pIR1SAT (squares), 10^6^ *L. major* NTPD1 null mutant + pIR1SAT (closed triangles) or 10^6^ *L. major* NTPD1 null mutant + pIR1SAT-*ntpd1* (open triangles). Error bars represent S.E.M. (*n* = 5). D and E. Mice were infected with either 10^5^ wild type *L. major* (squares) or 10^5^ *L. major* NTPD2 null mutant (circles) parasites and lesion scores monitored weekly. Error bars represent S.E.M. (*n* = 5). No significant difference in lesion size was observed between strains at any individual time point (*P*>0.05, two-way ANOVA).

To investigate whether LmNTPDase1 or 2 is required for virulence in the mammalian host, we tested the ability of *L. major* Δ*ntpd1* and *Δntpd2* to induce lesions in susceptible BALB/c mice. Promastigote stages of the *L. major* NTPD1 null mutant exhibited a marked and highly reproducible delay in lesion development. This delay was largely abrogated by complementation of the null mutant by insertion of a full-length *ntpd1* gene in the highly-transcribed ribosomal *ssu* locus [52]. Interestingly, no delay in lesion development was observed when amastigote stages of the NTPD1 null mutant were used to initiate the infection (Fig. 3A–C). Together, these studies demonstrate that NTPD1 is required during the early stages of promastigote infectivity, but has limited function in production of lesions following amastigote infection.

In contrast to the NTPD1 null mutant, the NTPD2 null mutant exhibited a virulence phenotype in BALB/c mice that was indistinguishable from wild type parasites, regardless of whether promastigotes or amastigotes were used to initiate infection (Fig. 3D and 3E). Infections were repeated a number of times and it is possible that these parasites have adapted to loss of NTPD2. Regardless, these results suggest that NTPD2 is not required for virulence in the mammalian host. Lesion development within the mouse reflects both parasite replication and the host response, and our results do not rule out an alteration in parasite replication levels between wild type and the NTPD2 null mutant. However the ability to cause disease, as measured by lesion size, was unchanged between the two strains.

### The *L. major* NTPD1 null mutant is defective in LPG elongation

**Figure 4:**
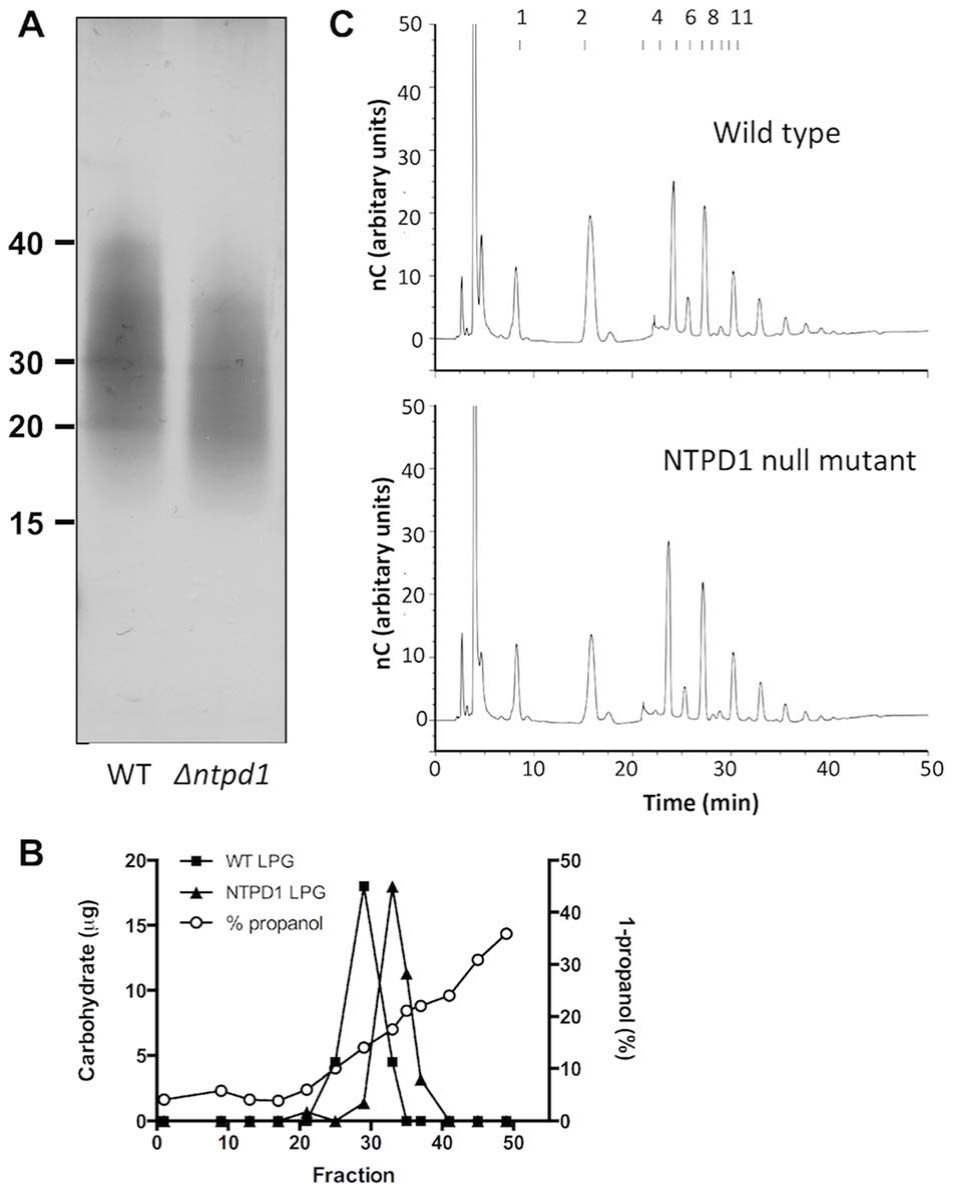
Analysis of purified LPG. A. LPG extracted from *L. major* wild type (WT) and *L. major Δntpd1* after SDS-PAGE and silver staining, demonstrating a clear difference in apparent molecular weight. Numbers indicate approximate molecular weight markers (kDa). B. Elution profile during octyl-Sepharose chromatography of LPG extracted from wild type *L. major* (squares) and the NTPD1 null mutant (triangles). LPG content was determined by orcinol staining [3:5-dihydroxy-toluene, BDH; 0.2%(w/v) in 10% H_2_SO_4_ and 50% ethanol], followed by colour development at 100°C and comparison to a known standard. The 1-propanol gradient concentration (open circles) was measured refractometrically. C. Fractionation of the dephosphorylated repeat units of LPG from wild-type and NTPD1 null mutant promastigotes. LPG was purified by octyl-Sepharose chromatography, depolymerised with 40 mM trifluoroacetic acid (8 min, 100°C) and dephosphorylated with calf intestinal alkaline phosphatase. The repeat units were desalted by passage over a mixed bed ion exchange column and chromatographed by HPAEC. The numbers at the top of the profile represent the elution positions of dextran oligomers (number of glucose units).

By analogy with the function of the Golgi-located yeast NTPDase, we predicted that NTPD1 may be involved in regulating the recycling of sugar-nucleotides in the Golgi lumen and hence glycosylation pathways [48], [49]. This hypothesis was further supported by the delayed lesion virulence phenotype of the NTPD1 null mutant, which is reminiscent of that seen previously for *L. major* mutant parasites that lack the major surface glycoconjugate, LPG [5], [53]. While LPG has multiple roles in the sandfly vector, it is only required for the early stages of promastigote infectivity in the mammalian host. LPG is not required for survival or growth of intracellular amastigotes, and LPG mutant parasites that survive the innate immune responses of the mammalian host can subsequently induce normal lesions [4], [5], as observed for the NTPD1 null mutant. To assess whether the *L. major* NTPD1 null mutant was defective in LPG biosynthesis, the de-lipidated wild type and mutant promastigotes were extracted in 9% 1-butanol and the lipoglycoconjugates purified by octyl-Sepharose chromatography [41]. The NTPD1 null mutant produced comparable levels of LPG as wild type parasites (Fig. 4A). As expected, both LPG preparations were visualized as smears on SDS-PAGE gels, reflecting heterogeneity in the length of the phosphoglycan chains that comprise the major portion of the LPG [42]. However, the LPG isolated from null mutant promastigotes reproducibly exhibited a lower average molecular weight on the SDS-PAGE gels (Fig. 4A) and eluted later from the octyl-Sepharose column (Fig. 4B), indicating shorter average chain length and/or reduced side chain branching. To distinguish between these possibilities, the LPG prepared from wild type and Δ*ntpd1* promastigotes was depolymerized with mild acid treatment (40 mM TFA, 100°C, 8 min) and dephosphorylated prior to analysis by HPAEC. Both LPG preparations had essentially identical oligosaccharide repeat unit profiles (Fig. 4C). Furthermore, LC/MS analysis of the released PI lipid moieties showed that both wild type and mutant LPG contained identical very long chain (C24:0, C26:0) alkylglycerol moieties. Collectively, these structural analyses suggest that the faster SDS-PAGE mobility of LPG isolated from the NTPD1 null mutant reflects decreased phosphoglycan chain elongation, rather than altered side chain additions or increased hydrophobicity in the lipid anchor.

**Figure 5:**
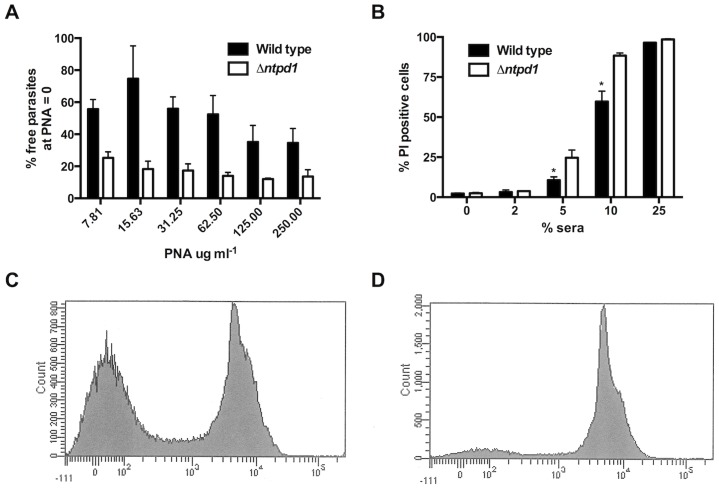
Truncated LPG synthesis by *L. major NTPD1* null mutants alters parasite biology. A. The number of free-swimming parasites observed following incubation with varying concentrations of PNA, expressed as a percentage of the number of free-swimming parasites observed in the absence of PNA. Compared to wild type *L. major* (black columns), significantly less unbound *L. major Δntpd1* (white columns) were observed at lower concentrations of PNA (*P<0.05), a trend that continued even at high concentrations of PNA. Data represents a minimum of three biological repeats. B. Percentage of parasites that were PI positive (indicating lysis) following incubation with varying concentrations of human sera. Significantly more *L. major Δntpd1* (white columns) were lysed when compared to wild type *L. major* at sera concentrations of 5 and 10. C and D. Representative flow cytometric analysis of parasites incubated with 10% human sera, demonstrating two populations of cells (lysed and intact) for wild type *L. major* (C), but only one major fluorescent (lysed) cell population for *L. major Δntpd1* (D). Data represents three biological repeats.

Expression of shorter LPG chains on the surface of the NTPD1 null mutant would be expected to lead to increased surface binding by the lectin, peanut agglutinin (PNA). PNA binds terminal β-Gal residues in the LPG side chains and intensity of binding is regulated by the abundance of β-Gal side chain, the extent to which these side chains are capped with arabinose and the overall length of the LPG [43]. Paradoxically, promastigotes expressing long LPG chains form surface aggregates in which LPG epitopes become cryptic and therefore bind less PNA. NTPD1 null mutant promastigotes were more effectively agglutinated than wild type promastigotes when harvested at the same stationary growth phase (Fig. 5A). Given that both wild type and mutant produce LPG with essentially identical side chain compositions (Fig. 4C), these results are consistent with the NTPD1 null promastigotes having a defect in LPG elongation.

### The *L. major* NTPD1 null mutant is more susceptible to complement lysis

To assess whether the defect in LPG chain elongation was physiologically significant, stationary phase wild type and NTPD1 null promastigotes were incubated with increasing concentrations of human serum. The complement resistance of *L. major* promastigotes has previously been shown to be highly dependent on LPG chain length and the formation of a thick protective surface glycocalyx [5]. NTPD1 null mutant promastigotes were significantly more sensitive to serum lysis than wild type parasites (Fig. 5B–D). In particular, FACS analysis of PI-stained parasites, showed ∼2-fold increased sensitivity at 5% serum concentrations (Fig. 5B). Collectively, these results provide strong evidence that loss of Golgi NTPDase results in less efficient elongation of LPG in virulent stationary phase promastigotes, leading to increased susceptibility to complement lysis and a marked delay in lesion development.

## Discussion

The genomes of many parasitic protozoa encode one or more NTPDases, which have been implicated in various host-parasite processes [6]–[9], [19]. However, the function of these enzymes in pathogenesis has not been rigorously defined using genetic approaches. In this study we have defined the subcellular localization and function of two clearly defined NTPDase enzymes in *L. major*. Both proteins are predicted to contain the five ACR domains that characterize NTPDases and to be constitutively transcribed in the two major life cycle stages. Based on analysis of GFP fusion proteins, we provide evidence that NTPD1 is primarily targeted to the Golgi apparatus, while NTPD2 is secreted into the extracellular milieu. We propose that NTPD1 has an important role in regulating glycosylation pathways in the Golgi apparatus as loss of NTPD1 resulted in a defect in LPG elongation in stationary phase promastigotes. Although the overall decrease in LPG chain length in the NTPD1 null mutant was modest, it was associated with significantly increased sensitivity to complement lysis and a conspicuous delay in lesion development when promastigotes were used to initiate infection. A similar lag in lesion development was not observed when NTPD1 null mutant amastigotes were used to initiate infection, consistent with the defect being associated with a promastigote-specific virulence factor such as LPG. The similarity between the virulence phenotype of the NTPD1 null mutant and previously generated *L. major* LPG mutants in which assembly of the entire phosphoglycan chain has been disrupted is striking [4], [53], and strongly suggests that LPG chain elongation during stationary phase is both critical for promastigote virulence, and likely to underlie the major function of this glycoconjugate during the early stages of infection in the mammalian host.

**Figure 6:**
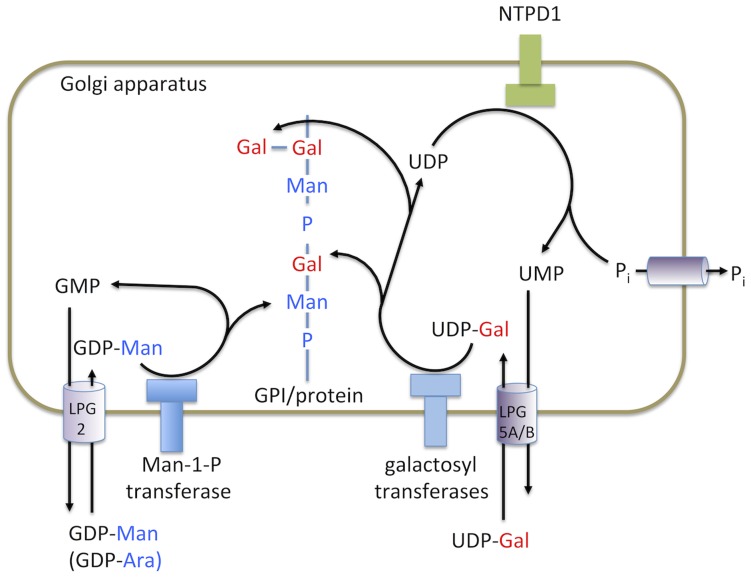
Proposed model for the role of NTPD1 in Golgi nucleotide-sugar transport and LPG synthesis. UDP-galactose and GDP-mannose/GDP-arabinose are transported into the Golgi via transporters LPG5A/LPG5B [37] and LPG2 [65] respectively. Galactose and mannose-phosphate are cleaved for use in phosphoglycan synthesis. Following cleavage, GMP is exchanged for GDP-mannose transport into the lumen. In the case of UDP, hydrolysis to UMP is catalyzed by NTPD1, allowing efficient ongoing transport of UDP-galactose into the Golgi lumen.

*S. cerevisiae* expresses two NTPDases, GDA1 and YND1, that are targeted to the Golgi apparatus with their catalytic domains orientated into the lumen [48], [49], [54]. These enzymes have been shown to hydrolyze NDP nucleotides to the corresponding NMP nucleotide, which is then used as the counter ion to import sugar nucleotides from the cytoplasm into the Golgi lumen. NTPDase-mediated hydrolysis of NDPs is thus critical for maintaining luminal levels of a range of sugar nucleotides that are used by Golgi glycosyltransferases [55]. In *Leishmania*, the Golgi apparatus contains enzymes required for the assembly and elongation of complex phosphoglycans on GPI anchor precursors, as well as a number of cell surface and secreted proteophosphoglycans (PPGs). All of these phosphoglycans contain the biosynthetic repeat unit, Galβ1-4Manα1-PO_4_, which is assembled by sequential transfer of Manα-1phosphate and galactose to the growing phosphoglycan chain by GDP-Man and UDP-Gal-dependent Golgi glycosyltransferases, respectively. The reactions catalyzed by the UDP-Gal dependent galactosyltransferases generate UDP, which would need to be converted to UMP by a NTPDase activity in order to sustain continued import of UDP-Gal into the Golgi lumen (Fig. 6). In contrast, the GDP-Man dependent Man-1-PO_4_-transferase(s) generate GMP, rather than GDP, and this NMP could be used to drive import of GDP-Man independent of the NTPDase activity. Thus the Golgi NTPDase is likely to be exclusively required for the galactosyltransferase-mediated reactions and not the GDP-Man-dependent Man-1-PO_4_ reactions. The fact that we see a specific defect in LPG chain elongation, but not in side chain modifications in the NTPDase mutant implies that β1-4-galactosyltransferase involved in assembly of the repeat unit backbone is more sensitive to depletion of UDP-Gal in the Golgi lumen than the β1-3galactosyltransferases that add additional galactose residues to the repeat unit backbone. At present, essentially nothing is known about the mechanisms that regulate LPG elongation, notwithstanding the importance of this process during the differentiation of rapidly dividing promastigotes to non-dividing, hypervirulent metacyclic promastigotes in culture and in the sandfly vector. Our findings raise the possibility that the changes in the availability of sugar nucleotides, either through changes in the activity/expression levels of Golgi membrane transporters or the luminal orientated NTPD1, could play an important role in this respect.

In contrast to NTPD1, deletion of NTPD2 had no measurable impact on the growth of *L. major* promastigotes *in vitro* or *in vivo*. As NTPD2 was secreted into the medium, it is unlikely that the absence of a detectable LPG or virulence phenotype in the NTPD2 mutant reflects redundancy between the two NTPDases. One possibility is that secreted NTPDase2 is primarily required for salvage of extracellular purines. *Leishmania* are purine auxotrophs but express a number of surface nucleotidases, acid phosphatases, nucleotide/nucleoside/purine base transporters, as well as intracellular enzymes involved in interconverting different purine intermediates [56]. This robust network of redundant purine salvage pathways could account for the absence of a conspicuous phenotype in the NTPD2 null mutant.

A recent study has suggested that *L. braziliensis* LbNTPDase1 is localized on the cell surface of promastigotes [21], and that opsonization with a polyclonal antibody directed to this protein was cytotoxic. Using this antibody, the authors also suggested that LbNTPDase1 may be additionally targeted to the mitochondria, cytoplasmic vesicles, kinetoplast and nucleus. It is possible that the *Leishmania* NTPDase1 homologues are targeted to different subcellular localizations in a species-specific manner and perform different functions. Further work to validate the specificity of the LbNTPDase1 polyclonal antibodies and/or determination of tagged proteins would be of interest.

Previous work demonstrated variation in the level of ecto-nucleotidase activity between *Leishmania* species [57]. Activity in *L. major* was lower than that observed for *L. amazonensis*, which was also more virulent in the mouse model used in the study, suggesting that the role of NTPDases in the disease process could differ between species of *Leishmania*. However, this study did not demonstrate that the observed ecto-nucleotidase activity was linked to *ntpd* gene expression, and the activity may relate to other enzymes. The same study also utilised Western blot analysis, using polyclonal antibody against *T. cruzi* NTPDase, to detect a band corresponding to the predicted size of NTPDase1 in *L. amazonensis*, but failed to identify a similar band in *L. major*. This may be due to failure of the antibody to recognize the *L. major* NTPDase, but could also suggest the natural level of expression of NTPDase1 in *L. major* is lower. However, in light of our findings that LmNTPDase1 localises to the Golgi apparatus, it is unlikely that lower expression of LmNTPDase1 would result in lower ecto-nucleotidase activity of *L. major*. Future studies taking defined genetic approaches to study NTPDases in other species of *Leishmania* would be extremely valuable in both defining their function, and in elucidating the value of this class of enzymes as a potential therapeutic target in *Leishmania*.

It is also important to recognize that a number of studies have implicated general surface-located hydrolysis of ATP, ADP (and sometimes other NTPs and NDPs) in the virulence of both *Leishmania* and a number of other parasites [18], [19], [58]–[62]. This observed activity has often been assumed to be due to the presence of NTPDases. However, our data raise the possibility that other classes of parasite enzymes are responsible for the observed activity and play a role in pathogenesis themselves. For example, a known NTPDase inhibitor, ARL67156, only inhibits 30% of observed ecto-ATPase activity of *T. cruzi* [6], suggesting that investigation of other classes of enzymes would also be worthwhile. It may be that a combinatorial approach is required, and that inhibition of two or more surface enzymes could be successful in treating disease.

In conclusion, this work considerably expands our knowledge of the role of *Leishmania* NTPDases in host-parasite interactions. We show for the first time that parasite NTPDases can be targeted to the Golgi, and play an important role in regulating the assembly of surface virulence factors. Unexpectedly, and notwithstanding previous studies suggesting that secreted NTPDases may have essential roles in purine acquisition, and/or host or parasite purinergic signalling, loss of the secreted NTPD2 had no discernible affect on promastigote or amastigote infectivity in mice. These studies highlight the importance of exploiting genetic approaches whenever possible in investigating the function of these enzymes in host-parasite interactions.

## Supporting Information

S1 Fig **PCR confirmation of deletion of** ***ntpd*** **genes in** ***L. major*** **.** A. Schematic demonstrating the location of primers used in polymerase chain reaction (PCR) analysis (see S1 Table for specific sequences). Dotted line indicates region of chromosome included in plasmid used to generate mutant. Arrows represent approximate location of primers, either upstream of this region, within the resistance (R) gene or within the specific *ntpd* gene. B. PCR products indicating the presence or absence of the *ntpd*1 gene (*ntpd*1) and the correct integration of the puromycin (pur) and hygromycin (hyg) cassettes onto the chromosome in place of the *ntpd*1 gene. Template for each reaction was either wild type *L. major* (W), deionised sterile water (-) or the *L. major* NTPD1 null mutant (M). Expected band size for the *ntpd*1 PCR was 1230 base pairs (bp), for the pur integration PCR was 1276 bp and for the hyg integration PCR was 1468 bp. Results clearly indicate the complete absence of the *ntpd*1 gene from the deletion mutant and the integration of the two resistance genes in its place, and confirm the absence of any additional alleles encoding *ntpd*1 in the *L. major ntpd*1 deletion mutant. C. Polymerase chain reaction products indicating the presence or absence of the *ntpd*1 gene (*ntpd*2) and the correct integration of the puromycin (pur) and bleocin (ble) cassettes onto the chromosome in place of the *ntpd*2 gene. Template for each reaction was either wild type *L. major* (W), deionised sterile water (-) or the *L. major* NTPD2 null mutant (M). “x” indicates and empty lane. Expected band size for the *ntpd*2 PCR was 2047 base pairs, for the pur integration PCR was 1081 base pairs and for the ble integration PCR was 1147 base pairs. Results clearly indicate the complete absence of the *ntpd*2 gene from the deletion mutant, the integration of the two resistance genes in its place, and confirm the absence of any additional alleles encoding *ntpd*2 in the *L. major ntpd*2 deletion mutant. PCR analysis was performed at a number of time points during culture, as well as before and after mouse infection, and typical results are presented.
(TIFF)

S1 Table **Primer sequences used in genetic manipulation of** ***L. major*** **and screening of drug resistant parasite lines for NTPD null mutants.**
(DOCX)

S2 Table **Accession numbers for sequences used to generate** **Fig. 1A and B** **.**
(DOCX)
