# Supplementary figures and images for: Golgi-Located NTPDase1 of Leishmania major Is Required for Lipophosphoglycan Elongation and Normal Lesion Development whereas Secreted NTPDase2 Is Dispensable for Virulence
Source: PLoS Negl Trop Dis. 2014 Dec 18;8(12):e3402. doi: 10.1371/journal.pntd.0003402 (PMC4270689; doi:10.1371/journal.pntd.0003402)

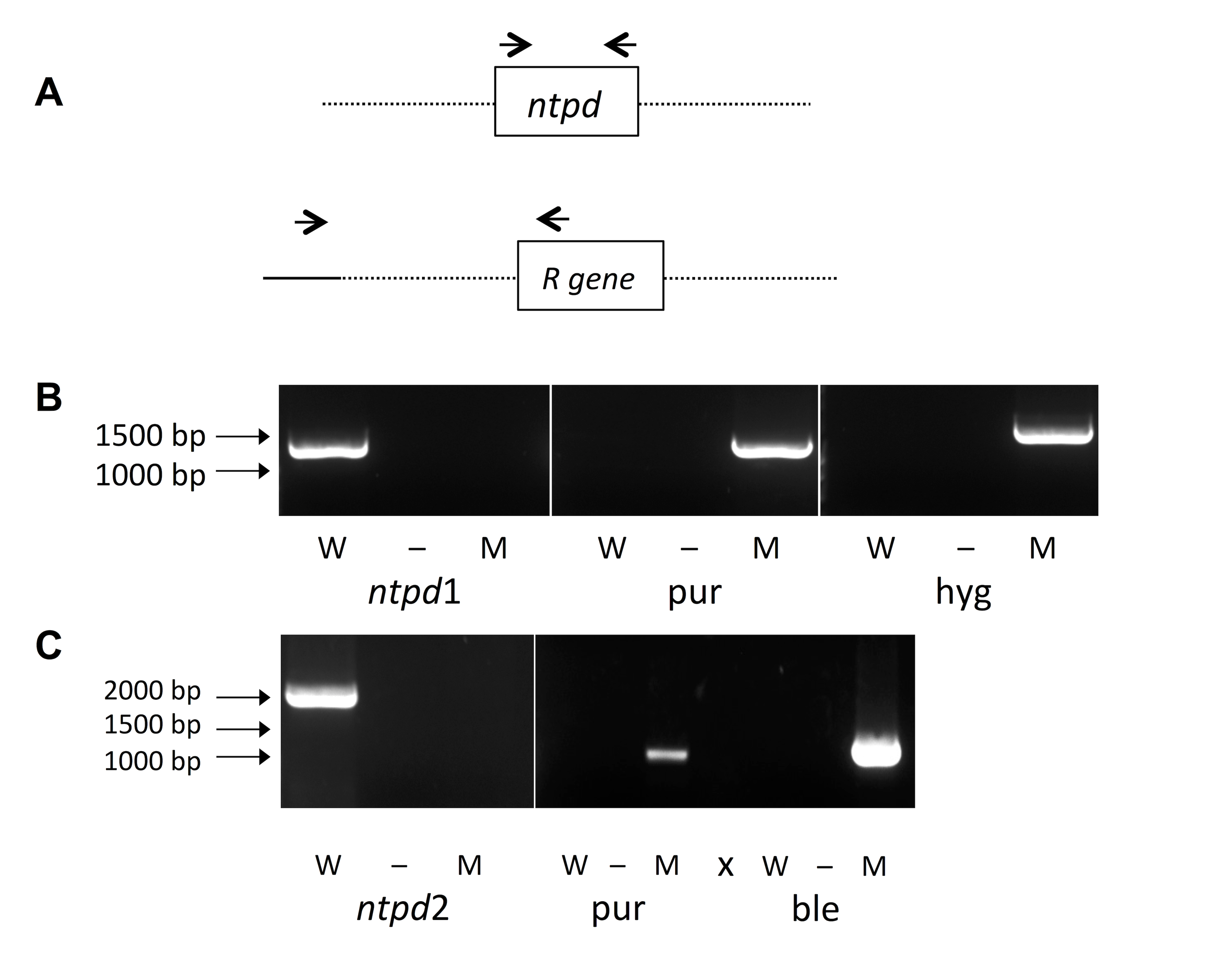

Supplement: S1 Fig — PCR confirmation of deletion of ntpd genes in L. major . A. Schematic demonstrating the location of primers used in polymerase chain reaction (PCR) analysis (see S1 Table for specific sequences). Dotted line indicates region of chromosome included in plasmid used to generate mutant. Arrows represent approximate location of primers, either upstream of this region, within the resistance (R) gene or within the specific ntpd gene. B. PCR products indicating the presence or absence of the ntpd1 gene (ntpd1) and the correct integration of the puromycin (pur) and hygromycin (hyg) cassettes onto the chromosome in place of the ntpd1 gene. Template for each reaction was either wild type L. major (W), deionised sterile water (-) or the L. major NTPD1 null mutant (M). Expected band size for the ntpd1 PCR was 1230 base pairs (bp), for the pur integration PCR was 1276 bp and for the hyg integration PCR was 1468 bp. Results clearly indicate the complete absence of the ntpd1 gene from the deletion mutant and the integration of the two resistance genes in its place, and confirm the absence of any additional alleles encoding ntpd1 in the L. major ntpd1 deletion mutant. C. Polymerase chain reaction products indicating the presence or absence of the ntpd1 gene (ntpd2) and the correct integration of the puromycin (pur) and bleocin (ble) cassettes onto the chromosome in place of the ntpd2 gene. Template for each reaction was either wild type L. major (W), deionised sterile water (-) or the L. major NTPD2 null mutant (M). “x” indicates and empty lane. Expected band size for the ntpd2 PCR was 2047 base pairs, for the pur integration PCR was 1081 base pairs and for the ble integration PCR was 1147 base pairs. Results clearly indicate the complete absence of the ntpd2 gene from the deletion mutant, the integration of the two resistance genes in its place, and confirm the absence of any additional alleles encoding ntpd2 in the L. major ntpd2 deletion mutant. PCR analysis was performed at a nu [file pntd.0003402.s001.tiff]
