# Supplementary material for: Golgi-Located NTPDase1 of Leishmania major Is Required for Lipophosphoglycan Elongation and Normal Lesion Development whereas Secreted NTPDase2 Is Dispensable for Virulence
Source: PLoS Negl Trop Dis. 2014 Dec 18;8(12):e3402. doi: 10.1371/journal.pntd.0003402 (PMC4270689; doi:10.1371/journal.pntd.0003402)
Supplement: S1 Table — Primer sequences used in genetic manipulation of L. major and screening of drug resistant parasite lines for NTPD null mutants. (DOCX) [file pntd.0003402.s002.docx]

**Table S1.**

| Primer Description | Primer Sequence |
| --- | --- |
| Forward primer (FP) for 5’ UTR of *ntpd1* | GGGGTACCCCGAAGCTGCCGCCCTG |
| Reverse primer (RP) for 5’ UTR of *ntpd1* | CCGCTCGAGCCCATCCTGACACACAACAG |
| FP for 3’ UTR of *ntpd1* | CGGGATCCCCACCGCCCCTGAGGAAAC |
| RP for 3’ UTR of *ntpd1* | CGAGCTCCCCTCGTCGTTTTTTGTTGAGGAG |
| FP for 5’ region of *ntpd2* | CCCAAGCTTGAGTCGCGTCGTTCTTGCCTTC |
| RP for 5’ region of *ntpd2* | CCGCTGGGATCCGAATTCGTCGCTGAGCTCACTGCCG |
| FP for 3’ UTR of *ntpd2* | GAATTCGGATCCCAGCGGAAACGGCATTTGGGCTGG |
| RP for 3’ UTR of *ntpd2* | ATAAGAATGCGGCCGCGAGAAGCCTTTGCCGTCAC |
| FP for *ntpd1* cloning into pXG-GFP^+^ | CCCGGATCCACCATGCGTCCGTACTCCTCG |
| RP for *ntpd1* cloning into pXG-GFP^+^ | CGATATCTTCCATCTTGAGCAGGAAGG |
| FP for *ntpd2* cloning into pXG-GFP^+^ | CCCGGATCCTTAGGTAAGAGAGAGGAGTGAG |
| RP for *ntpd2* cloning into pXG-GFP^+^ | CGATATCGGTAGAGAGGAGTGAG |
| FP for mCherry cloning into pXGSAT | TCCCCCGGGAGATCTCGATCGGCCACCATGGTGAGCAAGGGCGAG |
| RP for mCherry cloning into pXGSAT | CGGGATCCTTACCATGGGATATCCTTGTACAGCTCGTCCATGC |
| FP for *LPG1* cloning into pXGSAT-mCherry | TCCCCCCGGGGCCACCATGGCGCCTCGTCGCTG |
| RP for *LPG1* cloning into pXGSAT-mCherry | GAAGATCTGCTAGGATCAACAGCAAAGTCCAG |
| RP for *ntpd1* PCR | GGGATTCATGCATCAAAGCCCTTTGTTCTC |
| FP for *ntpd1* PCR | TGTCATCGTCGTCCTTGTAGTCTTCCATCTTGAGCAGGAAG |
| RP specific to *hyg* gene | GCACGAGATTCTTCGCCCTC |
| FP on *L. major* chromosome used to confirm integration of resistance cassettes into *ntpd*1 locus | CTGACTCCGCGGAAGAG |
| RP specific to *pur* gene | CGTGGGCTTGTACTCGGTC |
| FP on *L. major* chromosome used to confirm integration of resistance cassettes into *ntpd*2 locus | CCATCATGCCAATGACTGACG |
| RP specific to *ble* gene | GCACTGGTCAACTTGGCC |
| FP for *ntpd*2 gene | CCTATGCGGCCGCTGAACCACCATGAGTCGCGTCGTTCTTGC |
| RP for *ntpd*2 gene | CGTGTGCGGCCGCTTAGGTAAGAGAGAGGAGTGAG |

RP = reverse primer

FP = forward primer
