# Supplementary material for: Golgi-Located NTPDase1 of Leishmania major Is Required for Lipophosphoglycan Elongation and Normal Lesion Development whereas Secreted NTPDase2 Is Dispensable for Virulence
Source: PLoS Negl Trop Dis. 2014 Dec 18;8(12):e3402. doi: 10.1371/journal.pntd.0003402 (PMC4270689; doi:10.1371/journal.pntd.0003402)
Supplement: S2 Table — Accession numbers for sequences used to generate Fig. 1A and B . (DOCX) [file pntd.0003402.s003.docx]

**Table S2.**

| Protein Name | Sequence Identifier |
| --- | --- |
| LmNTPDase1 | XP_001681917.1 |
| LmNTPDase2 | XP_001681345.1 |
| LiNTPDase1 | XP_001464341 |
| LiNTPDase2 | XP_001463665 |
| LbNTPDase1 | XP_001562178 |
| LbNTPDase2 | XP_001562788 |
| LdNTPDase1 | CBZ32820.1 |
| LdNTPDase2 | CBZ32136.1 |
| LmxNTPDase1 | CBZ25018.1 |
| LmxNTPDase2 | CBZ24328 |
| Human CD39 | NP_001767 |
| TgNTPDase1 | Q27893 |
| TgNTPDase2 | Q27895 |
| NcNTPDase | BAA31454 |
| SnNTPDase | AAP88692 |
| PfNTPDase | XP_001348471.2 |
| TcNTPDase | AAS75599 |
| TbNTPDase1 | XP_847211.1 |
| TbNTPDase2 | XP_845817.1 |
| TvNTPDase1 | TVAG_063220 |
| TvNTPDase2 | TVAG_167570 |
| TvNTPDase3 | TVAG_397320 |
| TvNTPDase4 | TVAG_444510 |
| TvNTPDase5 | TVAG_351590 |
| *S. cerevisiae* GDA1 | NP_010872 |
| *S. cerevisiae* YND1 | EDN62971 |
